# Prévalence du surpoids et de l’obésité chez l’adolescent en milieu scolaire à Lubumbashi, République Démocratique du Congo

**DOI:** 10.11604/pamj.2019.32.49.15969

**Published:** 2019-01-28

**Authors:** Jacques Mbaz Musung, Emmanuel Kiyana Muyumba, Dophra Ngoy Nkulu, Placide Kambola Kakoma, Olivier Mukuku, Berthe Kon Mwad Kamalo, Clarence Kaut Mukeng, Christian Ngama Kakisingi, Francoise Kaj Malonga, Faustin Mukalenge Chenge, Oscar Numbi Luboya

**Affiliations:** 1 Département de Médecine Interne, Cliniques Universitaires de Lubumbashi, Université de Lubumbashi, République Démocratique du Congo; 2 Département de Médecine Interne, Hôpital Sendwe de Lubumbashi, Université de Lubumbashi, République Démocratique du Congo; 3 Institut Supérieur des Techniques Médicales de Lubumbashi, République Démocratique du Congo; 4 Ecole de Santé Publique, Université de Lubumbashi, République Démocratique du Congo; 5 Département de Pédiatrie, Cliniques Universitaires, Université de Lubumbashi, République Démocratique du Congo

**Keywords:** Surpoids, obésité, adolescent, prévalence, Lubumbashi, Overweight, obesity, adolescent, prevalence, Lubumbashi

## Abstract

**Introduction:**

Le surpoids et l’obésité au cours de l’adolescence constituent un problème préoccupant de santé publique à l’échelle mondiale en raison de leur retentissement potentiel sur la santé et de leur fréquence croissante. La présente étude avait pour objectif de déterminer la prévalence du surpoids et de l’obésité chez les adolescents scolarisés dans les établissements publics et privés à Lubumbashi, en République Démocratique du Congo.

**Méthodes:**

Il s’agissait d’une étude transversale menée auprès de 5.341 adolescents âgés de 10 à 19 ans, dont 2.858 (53,5%) filles et 2.483 (46,5%) garçons ont constitué notre échantillon. Pour chacun d’eux, nous avons mesuré le poids et la taille puis calculé l’indice de masse corporelle (IMC).

**Résultats:**

La moyenne du poids était de 43,78 ± 11,62 kg (soit 42,39 ± 12,11 kg pour les garçons et 44,95 ± 11,04 kg pour les filles), celle de la taille était de 151,30 ± 13,09 cm (soit 151,20 ± 14,64 cm pour les garçons et 151,38 ± 11,58 cm pour les filles) et celle de l’IMC était de 18,82 ± 3,15 kg/m^2^ (soit 19,39 ± 3,39 kg/m^2^ pour les garçons et 18,17 ± 2,71 kg/m^2^ pour les filles). La prévalence du surpoids était de 8% et celle de l’obésité était de 1%. Les filles étaient significativement plus touchées par le surpoids (10,7% filles contre 5% garçons) et l’obésité (1,5 % filles contre 0,4% garçons) que les garçons.

**Conclusion:**

Le surpoids et l’obésité chez les adolescents en milieu scolaire s’avèrent une réalité à Lubumbashi. La détermination de la prévalence du surpoids et de l’obésité pour cette catégorie d’âge au plan national est recommandable pour leurs préventions et prises en charges.

## Abstract (en)

**Introduction:**

overweight and obesity in adolescents are a major global public health issue due to their potential impact on health and increasing frequency. This study aims to determine the prevalence of overweight and obesity among adolescents attending public and private schools in Lubumbashi (DRC).

**Methods:**

we conducted a cross-sectional study of 5341 adolescents aged 10-19 years, 2858 (53.5%) girls and 2483 (46.5%) boys. Weight and height were measured for each adolescent and then body mass index (BMI) was calculated.

**Results:**

the average weight was 43,78 ± 11.62 kg (42,39 ± 12.11 kg for boys and 44.95 ± 11.04 kg for girls), the average height was 151,30 ± 13,09 cm (151.20 ± 14.64 cm for boys and 151,38 ± 11.58 cm for girls) and BMI was 18,82 ± 3.15 kg/m² (19.39 ± 3.39 kg/m² for boys and 18.17 ± 2.71 kg/m² for girls). The prevalence of overweight was 8% while that of obesity was 1%. The girls were significantly more affected by overweight (10.7% girls against 5% boys) and obesity (1.5% girls against 0.4% boys) than the boys.

**Conclusion:**

overweight and obesity in school environment are a reality in Lubumbashi. The prevalence of overweight and obesity in this age group category should be determined in a national evaluation plan in order to implement preventive and therapeutic strategies.

## Introduction

Le surpoids et l’obésité au cours de l’adolescence constituent un problème préoccupant de santé publique à l’échelle mondiale en raison de leur retentissement potentiel sur la santé et de leur fréquence croissante [1]. L’Organisation Mondiale de la Santé (OMS) a déclaré que le surpoids était l’un des dix principaux risques pour la santé dans le monde et l’un des cinq premiers dans les pays développés [2]. L’obésité est un facteur de risque bien connu pour divers problèmes de santé chroniques tels que les maladies cardiovasculaires, l’hypertension, les accidents vasculaires cérébraux, le diabète sucré de type 2, l’ostéoarthrite et certains cancers. Ces conditions entrainent non seulement une qualité de vie réduite étant donné leur caractère prolongé, mais également à une mort prématurée [3]. L’obésité est épidémique dans le monde en affectant aussi bien les adultes que les enfants et les adolescents [4]. Plus de la moitié de la population adulte serait en surpoids ou obèse en 2030 [5]. Une augmentation de la prévalence du surpoids et de l’obésité a été observée chez les adultes, cela est également constaté chez les adolescents au cours des dernières décennies [6]. Les estimations mondiales de la prévalence du surpoids et de l’obésité chez les enfants et adolescents faites en 2004 avaient montré qu’environ 150 à 160 millions d’enfants d’âge scolaire (5 à 17 ans) dans le monde étaient en surpoids, dont 35 à 40 millions étaient obèses [7]. L’obésité à l’adolescence est un facteur prédictif pour l’obésité à l’âge adulte et s’accompagne alors de risque de morbidité accrus chez les sujets ayant été en surpoids à l’adolescence, même chez ceux qui rejoindront un poids normal à l’âge adulte [8]. Burke *et al.* ont rapporté que les enfants qui étaient en surpoids ou obèses à l’âge de 9 ans étaient de 16 %, le reste à l’âge de 25 ans avec des proportions respectives de 24 % pour les adolescents de 12 ans, 34% pour ceux de 15 ans et 35% pour ceux de 18 ans (p < 0,001) [9]. Aux Etats-Unis, 25% des enfants et adolescents présentent un IMC situé entre le 90^e^ et le 97^e^percentile, et 11% sont considérés comme obèses, ce qui correspond à un IMC au-delà du 97^e^ percentile [10]. Autrefois associé uniquement aux pays développés, le surpoids et l’obésité sont maintenant répandus dans les pays en développement [11, 12]. On estime actuellement qu’en Afrique, 20 à 50% des populations urbaines sont classées en surpoids ou obèses et qu’en 2025, les trois quarts de la population obèse mondiale se trouveront dans des pays en développement [13]. Chez les adolescents Ethiopiens âgé de 10 à 19 ans, Teshome *et al.* avait rapporté une prévalence du surpoids et de l’obésité respectivement de 12.9 % et 2.7 % [14]. En Algérie, Fedala *et al.* dans une étude réalisée chez les adolescents âgés de 10 à 19 ans en milieu scolaire, avait trouvé des prévalences respectives d’obésité et du surpoids de 2,42% et 6,6% (chez les garçons) et de 0,54% et 2,16% (chez les filles) [15]. En Tunisie, 18.9% des adolescents sont en surpoids et 4% sont obèses [16]. En 2011, une étude menée au Sénégal auprès des adolescents âgés de 11 à 17 sans au sein des établissements publics et privés de Dakar, indiquait une prévalence de 9,3% d’obésité [17]. Très peu d’études ont été consacrées au surpoids et à l’obésité dans notre milieu; ainsi cette étude avait pour objectif de déterminer la prévalence de ces deux pathologies chez les adolescents en milieu scolaire dans la ville de Lubumbashi en République Démocratique du Congo (RDC).

## Méthodes

### Type et population d’étude

Il s’agissait d’une étude transversale dont la population d’étude était constituée d’adolescents âgés de 10 à 19 ans, régulièrement inscrits dans les écoles primaires et secondaires de la ville de Lubumbashi (deuxième ville de la RDC par son importance économique, politique, sociale et culturelle) aussi bien du secteur privé que public. Cette population avait été sélectionnée de manière aléatoire selon un plan d’échantillonnage stratifié en grappes à deux niveaux. Ainsi, ont été sélectionnés successivement un échantillon d’écoles (réparties par commune) puis un échantillon des classes dans chaque école sélectionnée et enfin tous les élèves (unités statistiques) inscrits à l’intérieur des classes sélectionnées étaient retenus comme adhérents à l’enquête. Les élèves âgés de moins de 10 ans ou de plus de 19 ans où ayant des données incomplètes, aberrantes ou absentes n’ont pas été inclus. Les données ont été recueillies par l’équipe médicale d’étude formée qui recevait une formation de recyclage au début de chaque phase de collecte de données. Au finish, notre échantillon était composé de 5.341 adolescents.

### Variables d’études

Nous avons étudié l’âge, le sexe, la taille, le poids et l’IMC. Le poids, exprimé en kilogramme, était mesuré à deux reprises, chez un sujet légèrement habillé, déchaussé, debout sur une balance médicale numérique de marque SECA (SECA 881 U, Allemagne). La balance était placée sur une surface stable et plane. La taille était mesurée, à deux reprises, avec une toise verticale, chez un sujet déchaussé, debout, avec les épaules et les hanches perpendiculaires à l’axe central, les talons contre le marchepied, les genoux joints, les bras relâchés le long du corps et la tête bien droite (tête regardant devant de façon à ce que le rebord inferieur des orbites soit dans le même plan horizontal que le conduit auditif externe). Pour les analyses statistiques, la moyenne de deux mesures du poids et de la taille étaient retenue. L’IMC avait été calculé en divisant le poids exprimé en kilogramme par le carré de la taille exprimée en mètre. Nous avons utilisé la définition de l’International Obesity Task Force (IOTF) [18] et en se basant sur les recommandations de l’European Childhood Obesity Group pour les études épidémiologiques de Rolland-Cachera [19] : 1) surpoids: valeurs de centiles de l’IMC par rapport au sexe qui concordaient avec le point de coupure de 25 kg/m^2^ à l’âge de 18 ans; 2) obésité: valeurs de centiles de l’IMC par rapport au sexe qui concordaient avec le point de coupure de 30 kg/m2 à l’âge de 18 ans.

### Analyses statistiques

Les données ont été analysées au moyen du logiciel SPSS 21.0. Les variables quantitatives étaient représentées sous forme de moyenne, de déviation standard (DS), d’intervalle de confiance à 95% (IC95%) alors que celles qualitatives étaient représentées sous forme d’effectifs (n) et de pourcentage (%). Pour faciliter les calculs, l’âge était décimalisé au moyen de la formule d’IRETON [20]. Le test de Student nous a permis d’établir la comparaison de la moyenne d’âge, du sexe, de la taille et de l’IMC. Le test de Khi-carré de Pearson a été utilisé pour évaluer l’association entre les variables qualitatives. La valeur de p < 0,05 était considérée comme statistiquement significative et un test bilatéral a été utilisé.

### Considérations éthiques

Un consentement éclairé écrit avait été obtenu auprès des parents ou tuteur des adolescents avant la participation à l’étude. L’approbation de la tenue de l’étude ainsi que les autorisations y afférentes étaient obtenues du Comité d’Ethique Médicale de l’Université de Lubumbashi (UNILU/CEM/027/2013-27 septembre 2013), du Ministère Provincial de l’Education du Katanga (N° 10.5/0209/CAB/MIN.PROV/ED.R.TE/KAT/2014-11 March 2014) et des responsables des écoles sélectionnées.

## Résultats

Sur 5.341 adolescents âgés de 10 à 19 ans enregistrés dans l’étude, 2.858 étaient des filles (53,5%) et 2.483 des garçons (46,5%). Nos résultats dans le Tableau 1, Tableau 2 et Tableau 3 montrent que le poids, la taille et l’IMC augmentaient en fonction de l’âge. Les filles avaient un IMC significativement plus élevé que les garçons dans toutes les tranches d’âges. En comparant les deux sexes, nous constatons que les moyennes de nos variables étaient statistiquement différentes (p < 0,0001): les moyennes du poids, de la taille et de l’IMC étaient respectivement de 43,78 ± 11,62 kg (soit 42,39 ± 12,11 kg pour les garçons et 44,95 ± 11,04 kg pour les filles), de 151,30 ± 13,09 cm (soit 151,20 ± 14,64 cm pour les garçons et 151,38 ± 11,58 cm pour les filles) et de 18,82 ± 3,15 kg/m^2^ (soit 19,39 ± 3,39 kg/m^2^ pour les garçons et 18,17 ± 2,71 kg/m^2^ pour les filles). La prévalence du surpoids dans notre étude était respectivement de 8% (dont 10,7% chez les filles contre 5% chez les garçons) et celle de l’obésité était de 1% (dont 1,5% chez filles contre 0,4% chez les garçons). En comparant ces différentes prévalences de surpoids et d’obésité entre les deux sexes, le test de Pearson montre des différences très hautement significatives traduisant que les filles étaient significativement plus en surpoids et obèses que les garçons (Tableau 4).

**Tableau 1:** Répartition du poids selon l’âge et le sexe

Age	Garçons		Filles		p
	N	Poids (kg) Moyenne (ET)	N	Poids (kg) Moyenne (ET)	
10 ans	391	30,3 (5,2)	377	31,8 (6,4)	0,000
11 ans	388	34,3 (6,8)	416	36,5 (7,8)	0,000
12 ans	380	36,5 (6,6)	386	40,4 (7,8)	0,000
13 ans	296	39,4 (7,5)	330	45,2 (8,0)	0,000
14 ans	257	45,5 (8,3)	354	49,6 (7,8)	0,000
15 ans	200	50,8 (8,2)	304	51,7 (7,5)	0,227
16 ans	156	55,8 (6,9)	249	53,4 (8,2)	0,002
17 ans	162	57,0 (6,7)	218	54,2 (7,3)	0,000
18 ans	141	58,0 (7,3)	139	55,2 (7,5)	0,002
19 ans	112	59,0 (7,6)	85	55,7 (7,3)	0,002

N: effectif ; ET: écart-type

**Tableau 2:** Répartition de la taille selon l’âge et le sexe

Age	Garçons		Filles		p
	N	Taille (en cm) Moyenne (ET)	N	Taille (en cm) Moyenne (ET)	
10 ans	391	135,8 (8,4)	377	137,5 (9)	0,005
11 ans	388	141,8 (8,9)	416	143,9 (9,7)	0,001
12 ans	380	145,2 (9,2)	386	147,9 (9)	0,000
13 ans	296	149 (10,2)	330	152,4 (8,9)	0,000
14 ans	257	155,9 (10,2)	354	156,1 (8,2)	0,827
15 ans	200	161,5 (9,7)	304	157,8 (8,6)	0,000
16 ans	156	166,6 (7,9)	249	159,6 (7,4)	0,000
17 ans	162	167,4 (8,3)	218	160,1 (7,4)	0,000
18 ans	141	168 (9,2)	139	159,9 (8,9)	0,000
19 ans	112	169 (9)	85	158,3 (8,7)	0,000

N: effectif ; ET: écart-type

**Tableau 3:** Répartition de l’IMC selon l’âge et le sexe

Age	Garçons		Filles		p
	N	IMC (en kg/m²) Moyenne (ET)	N	IMC (en kg/m²) Moyenne (ET)	
10 ans	391	16,4 (2,1)	377	16,7 (2,4)	0,028
11 ans	388	17,0 (2,3)	416	17,5 (2,8)	0,003
12 ans	380	17,3 (2,2)	386	18,4 (2,7)	0,000
13 ans	296	17,7 (2,3)	330	19,5 (3,3)	0,000
14 ans	257	18,7 (2,5)	354	20,4 (2,9)	0,000
15 ans	200	19,5 (2,6)	304	20,8 (3,2)	0,000
16 ans	156	20,1 (2)	249	21,0 (3,2)	0,000
17 ans	162	20,4 (2,4)	218	21,2 (2,9)	0,003
18 ans	141	20,6 (2,4)	139	21,7 (3,5)	0,004
19 ans	112	20,7 (2,1)	85	22,3 (3,2)	0,000

N: effectif ; ET: écart-type

**Tableau 4:** Prévalences du surpoids et de l’obésité en fonction de l’âge et du sexe chez les adolescents à Lubumbashi (RDC)

Age	Sexe	Effectif	Normal n (%)	Surpoids n (%)	p*	Obésité n (%)	p**
10 ans	Garçons	391	370 (94,6%)	18 (4,6%)	0,029	3 (0,8%)	0,683
	Filles	377	343 (91,0%)	32 (8,5%)		2 (0,5%)	
	Total	768	713 (92,8%)	50 (6,5%)		5 (0,7%)	
11 ans	Garçons	388	359 (92,5%)	28 (7,2%)	0,383	1 (0,3%)	0,071
	Filles	416	373 (89,7%)	37 (8,9%)		6 (1,4%)	
	Total	804	732 (91,0%)	65 (8,1%)		7 (0,9%)	
12 ans	Garçons	380	356 (93,7%)	24 (6,3%)	0,514	0 (0%)	0,085
	Filles	386	354 (91,7%)	29 (7,5%)		3 (0,8%)	
	Total	766	710 (92,7%)	53 (6,9%)		3 (0,4%)	
13 ans	Garçons	296	280 (94,6%)	14 (4,7%)	0,003	2 (0,7%)	0,204
	Filles	330	287 (87,0%)	37 (11,2%)		6 (1,8%)	
	Total	626	567 (90,6%)	51 (8,1%)		8 (1,3%)	
14 ans	Garçons	257	243 (94,5%)	13 (5,1%)	0,000	1 (0,4%)	0,488
	Filles	354	301 (85,1%)	50 (14,1%)		3 (0,8%)	
	Total	611	544 (89,0%)	63 (10,3%)		4 (0,7%)	
15 ans	Garçons	200	185 (92,5%)	14 (7,0%)	0,060	1 (0,5%)	0,113
	Filles	304	260 (85,5%)	37 (12,2%)		7 (2,3%)	
	Total	504	445 (88,3%)	51 (10,1%)		8 (1,6%)	
16 ans	Garçons	156	152 (97,4%)	4 (2,6%)	0,005	0 (0,0%)	0,035
	Filles	249	217 (87,2%)	25 (10,0%)		7 (2,8%)	
	Total	405	369 (91,1%)	29 (7,2%)		7 (1,7%)	
17 ans	Garçons	162	157 (96,9%)	3 (1,9%)	0,000	2 (1,2%)	0,398
	Filles	218	192 (88,0%)	25 (11,5%)		1 (0,5%)	
	Total	380	349 (91,8%)	28 (7,4%)		3 (0,8%)	
18 ans	Garçons	141	135 (95,8%)	5 (3,5%)	0,019	1 (0,7%)	0,053
	Filles	139	118 (84,9%)	15 (10,8%)		6 (4,3%)	
	Total	280	253 (90,4%)	20 (7,1%)		7 (2,5%)	
19 ans	Garçons	112	111 (99,1%)	1 (0,9%)	0,000	0 (0,0%)	0,250
	Filles	85	66 (77,6%)	18 (21,2%)		1 (1,2%)	
	Total	197	177 (89,9%)	19 (9,6%)		1 (0,5%)	
Total	Garçons	2483	2348 (94,6%)	124 (5,0%)	0,000	11 (0,4%)	0,000
	Filles	2858	2511 (87,8%)	305 (10,7%)		42 (1,5%)	
	Total	5341	4912(91,0%)	429(8,0%)		53(1,0%)	

* p-value de la comparaison de prévalence du surpoids entre les garçons et les filles;

** p-value de la comparaison de prévalence de l’obésité entre les garçons et les filles

## Discussion

L’étude du surpoids et de l’obésité chez les adolescents est d’un grand intérêt dans nos pays en développement dans lesquels ces deux facteurs de risque sont en augmentation [11, 12]. Environ 54% des sujets de notre échantillon étaient de sexe féminin. Ce résultat est similaire à celui rapporté en Algérie dans une étude réalisée en milieu scolaire [15]. Par contre, dans le travail de Satyajit Bagudai *et al.* [21] en inde, 63,1% des adolescents étaient des garçons. Cela montre une disparité de l’accès des jeunes filles à l’école. Le poids, la taille et l’IMC augmentaient en fonction de l’âge. La moyenne de l’IMC (18,82 ± 3,15kg/m^2^) est comparable à celle rapportée par Fedala N *et al.* [15] en Algérie (IMC 19,03 ± 3,77) tandis que les moyennes du poids (43,78 ± 11,62 kg) et de la taille (1,51±0,13 m) sont inférieures à celles rapportées par le même auteur (poids 48,42 ± 11,51 kg et taille 1,59 ± 0,11 m). La prévalence du surpoids et de l’obésité dans notre étude était respectivement de 8% et 1%. Nos résultats sont similaires à ceux rapportés par les différents chercheurs de pays en voie de développement. En Ethiopie, la prévalence du surpoids et de l’obésité chez les enfants de 5 à 19 ans à Addis-Abeba était respectivement de 7,6% et de 0,9% [22]. Ce constat a été observé par Mpembeni RN *et al.* en Tanzanie [23]. Dans une étude menée en Afrique du Sud [24], 7,8% des adolescents scolarisés et âgés de 10 à 15 ans étaient en surpoids ou obèses. Sebbani M avait trouvé le même résultat que le nôtre en ce qui concerne la prévalence du surpoids (8% de cas du surpoids) et non en ce qui concerne le résultat de l’obésité dont la prévalence était supérieure au nôtre (3% de cas d’obésité ) [25]. Tandis que Dekkeki IC, avait trouvé le résultat de la prévalence du surpoids inferieure au notre soit 5,1% selon une étude faite en milieu scolaire dans les écoles primaires publiques à Rabat, tandis que le résultat de la prévalence de l’obésité était supérieur au notre soit 3,6% [26].

Dans notre étude, la prévalence du surpoids et de l’obésité était inférieure par rapport à celles trouvées dans plusieurs études dont celle de Leung LC *et al.* menée dans une population d’adolescents chinois âgés de 6 à 18 ans, avait rapporté une prévalence de 11,2% pour le surpoids et de 2,9% pour l’obésité [27]. Dans une étude réalisée en Lithuanie chez les adolescents âgés de 12 à 17 ans, Dulskiene V *et al.* avait observé une prévalence du surpoids et de l’obésité respectivement de 12,1% et 2,47% [28]. Alors qu’au sud de l’Iran, Basiratnia M *et al.* avait rapporté une prévalence de 13% pour le surpoids et de 7% pour l’obésité chez les adolescents de milieu scolaire âgés de 11 à 17 ans [29]. A Lagos (Nigeria), chez les adolescents âgés de 15 à 19 ans, Oduwole AA *et al.* avait enregistré un taux de 13,8% de surpoids et de 9,4% et d’obésité [30].

Les prévalences du surpoids et de l’obésité dans notre étude est inférieure à celles rapportées dans certains pays développés comme les États-Unis [31], le Canada [32], la Grèce [33] et l’Italie [34] ayant des prévalences du surpoids allant de 22 à 36% et de l’obésité de 4,15 à 9,45%. En France, une enquête de prévalence portant sur un échantillon de 1.507 adolescents âgés de 11 à 17 ans menée dans le département des Hauts-de-Seine montrait que 17,6% des élèves étaient en surcharge pondérale (obésité incluse) [35]. Cette même enquête rapportait que les prévalences du surpoids étaient de 7,7% chez les filles et de 10% chez les garçons et celles de l’obésité étaient de 5,5% chez les filles et de 11,7% chez les garçons [35]. Prado C *et al.*indiquait qu’en Espagne, la surcharge pondérale était de l’ordre de 29% chez les adolescents âgés de 13 à 16 ans [36]. Plusieurs études affirment que le niveau socio-économique élevé est un facteur de risque d’obésité dans les pays en développement tandis qu’un niveau socio-économique bas est également un facteur de risque pour les pays développés [37-40]. Cependant, le développement industriel et le changement du mode de vie contribuent à une augmentation annuelle rapide de l’obésité en milieu rural et urbain [41]. La différence observée entre les prévalences du surpoids et de l’obésité chez les adolescents dans les différents pays serait due à plusieurs raisons: l’absence d’une définition univoque du surpoids et de l’obésité et aussi la différence de la population d’étude.

La distribution du surpoids et de l’obésité en fonction du sexe était respectivement de 10,7% chez les filles contre 5% chez les garçons pour le surpoids et 1,5% chez les filles contre 0,4% chez les garçons pour l’obésité. Avec une différence statistiquement significative (p = 0,000).Ceci pourrait être expliqué par le rôle de la puberté dans le développement de la masse grasse et son impact sur la surestimation de la surcharge pondérale chez les filles durant cette période [42]. Une étude réalisée en Afrique du Sud [43] sur les comportements à risque des jeunes âgés de 13 à 19 ans avait trouvé que 16,9% des participants étaient en surpoids dont 24,5% étaient des filles et 2,2% étaient de garçons et 4% étaient obèses parmi lesquels 6,9% étaient des garçons et 5,3% étaient des filles. A Sousse (Tunisie), Gaha avait trouvé que, comparés aux garçons, les filles étaient plus en surpoids (16,1 contre 11,6% ; p=0,004) et plus obèses (3,3% contre 2,8% ; p=0,89) [44]. Une autre étude réalisée toujours dans le même pays (près de 10 ans plus tard) avait montré que les prévalences du surpoids et de l’obésité avaient augmenté et étaient restées plus élevées chez les filles (20,7% et 4,6%) que chez les garçons (17,2% et 4,0%) [45].

Des résultats contradictoires concernant la répartition du surpoids et de l’obésité selon le sexe ont été observés dans différents pays, dont le Canada [32], la Grèce [33] et l’Italie [34] où la prévalence était plus élevée chez les garçons que chez les filles. Dans une population d’adolescents algériens âgés de 10 à 19 ans, en milieu scolaire, les garçons étaient plus en surpoids et obèses (6,6% et 2,42%) que les filles (2,16% et 0,54%) [15]. Bagudai S *et al.* [21] en Inde, avait fait la même observation sur 5.155 adolescents scolarisés âgés de 10 à 16 ans. Ces différences de prévalences du surpoids et de l’obésité notées entre les deux sexes dans ces différentes populations pourraient être influencées par plusieurs facteurs entre autre sociodémographiques, culturels et économiques.

## Conclusion

Le surpoids et l’obésité chez les adolescents s’avèrent une réalité en milieu scolaire à Lubumbashi. Les filles sont plus touchées que les garçons. En l’absence de données nationales, notre étude offre une estimation de la prévalence du surpoids et de l’obésité des adolescents dans notre contexte. Les chiffres apportés par les différentes études nous incitent à prendre conscience de ce fléau et rendent compte de l’importance du problème. Ainsi, nous recommandons aux pouvoirs publics à tous les niveaux de rendre opérationnel et/ou renforcer le programme de Médecine scolaire afin de permettre de réaliser le dépistage systématique et la prise en charge de ce fléau dans tous les établissements scolaires de la RDC.

### Etat des connaissances actuelles sur le sujet

- L’obésité est épidémique dans le monde et affecte aussi bien les adultes que les enfants et les adolescents;
- L’obésité est un facteur de risque pour les pathologies chroniques. Elle réduit la qualité de vie et causes de décès prématurés;
- Le surpoids et l’obésité à l’adolescence persistent à l’âge adulte.

### Contribution de notre étude à la connaissance

- Le surpoids et l’obésité chez les adolescents s’avèrent une réalité en milieu scolaire à Lubumbashi;
- Le poids, la taille et l’IMC augmentent avec l’âge pour les deux sexes;
- Les filles sont plus touchées que les garçons par le surpoids et l’obésité.

## Conflits d’intérêts

Les auteurs ne déclarent aucun conflit d'intérêts.
